# The Doctor's Christmas

**Published:** 1892-12-24

**Authors:** 


### The Doctor's Christmas.

For most of us Christmas means "peace and plenty"—truly a day of rest and gladness when the daily round and common task may be put aside. But there is always the seamy side of life, and we have not far to look to find those to whom this is the familiar side. This is an oft-told tale, but the sadness of human suffering should be brought home to us all the more forcibly by contrast with the idea of festivity and happiness that is associated in our minds with Christmas.

Well do we know that the sick and diseased find by painful experience that the inexorable goddess Hygeia exacts hard penalties for transgressing her laws, irrespective of times and seasons, and that our hospitals and infirmaries must care for our poorer brethren at Christmas as at all other times.

It is right that we should be reminded of these thousands of poor sick folk at our doors, but let us also recollect that the work of tending and caring for them, the necessity to cheer the despondent and weary patient falls mainly on the hard-worked doctor and sick nurse, who so often themselves feel the need for their Christmas rest and relaxation.

How hard it is to forget oneself when our duties necessitate a long round of visits to private and hospital patients. Mr. Marion Crawford so happily describes the feeling we have all experienced when he says: "There is a hidden instinct of a low and cowardly kind, but human nevertheless, which bids us turn away from spectacles of agony, whether harrowing or repulsive, until the good angel comes and whispers that we must trample on such coarse impulses and do our duty." To every one engaged in hospital work the good angel, our higher nature, speaks, and rarely speaks in vain. But the outside world little knows of the thousand noble deeds and the self-sacrifices that our hospitals could tell of, though now and then a brilliant career, suddenly terminated by devotion to the call of duty, may thrill the public and let in the light on deeds that are done almost as a matter of course.

It is a trite saying that the medical profession do more gratuitous work for the public than any other profession, but it is taken far too much for granted by many of us who might do a great deal to help and encourage those who are brought so constantly face to face with such pitiful spectacles of human suffering.

But though Christmas may bring but little pleasure to the busy practitioner, the hospital physician, and the miserably paid parish doctor, it should give much happiness. "The aim of the ignorant is pleasure, the pursuit of the wise happiness, and pleasure is but the refreshment that cheers us in the pursuit of happiness."

Yes, doctor, if virtue has gone out of you, happiness at any rate, may be yours on this Christmas morn. To-day especially be the true physician to whom "there is a sanctity in the sick chamber. At its threshold all the more human passions quit their hold of him; love there would be profanation. The grief permitted to others he is denied, he must enter that room with a calm intelligence. If he allow aught to dim the keen glance of science he is unfit for his mission there. To him youth and age, beauty and deformity, poverty and wealth, guilt and innocence, merge themselves into one common attribute, human suffering appealing to human skill."

To go about doing good is delightful in the abstract but those alone who have tried it know that temper and good nature are often sorely tried, yet we cannot be tempted to treat lightly the claims of the meanest if we bear in mind the lines on

